# The impact of season, temperature, and direct normal irradiance on IVF pregnancy outcomes: a retrospective cohort study

**DOI:** 10.1007/s00484-025-02951-2

**Published:** 2025-06-18

**Authors:** Chao Wang, Jiehua Chen, Zhong Lin, Li Shi, Qiuyan Ruan, Jiamin Long, Yanping Lao, Xiangli Niu

**Affiliations:** 1 The Reproductive Hospital of Guangxi Zhuang Autonomous Region, Nanning, 530000 Guangxi China; 2 Department of Microbiology, School of Preclinical Medicine, Guangxi Medical University, Nanning, 530021 Guangxi China; 3 The Reproductive Hospital of Guangxi Zhuang Autonomous Region, No. 3 Longyuan Road, Nanning, 530000 Guangxi China

**Keywords:** Season, Temperature, In vitro fertilization, Clinical pregnancy

## Abstract

*Objective*. This study assessed associations between season, temperature, direct normal irradiance (DNI), and clinical pregnancy outcomes in first fresh in vitro fertilization (IVF) cycle. *Methods*. We conducted a retrospective cohort study (June 2021-October 2023) of 1179 patients undergoing fresh IVF cycles. Inclusion criteria required age ≤ 45 years, first-time fresh embryo transfer cycles (long/antagonist protocol), excluding preimplantation genetic testing, frozen cycles, or incomplete data. Temperature and DNI were recorded from gonadotropin (Gn) initiation to oocyte retrieval. Binary logistic regression assessed seasonal impacts on clinical pregnancy, while restricted cubic splines (RCS) independently modeled temperature/DNI effects. *Results*. Compared to winter, clinical pregnancy likelihood was 1.74-fold higher in spring (95% CI:1.11–2.71, *P* = 0.015) and 1.53-fold in summer (95% CI:1.02–2.30, *P* = 0.042). Stratified analysis revealed a 2.02-fold increased likelihood in summer cycles using the long protocol (95% CI:1.07–3.82, *P* = 0.031), whereas no seasonal variation was observed in antagonist protocol. RCS regression analysis indicated a nonlinear relationship between temperature and clinical pregnancy outcomes, which was confirmed in the long protocol group. No significant correlations were observed with DNI or in patients treated with the antagonist protocol. *Conclusions*. Season and ambient temperature significantly impact IVF clinical pregnancy, especially in the long protocol, with the highest increase in pregnancy rates observed in summer, as well as within the 26.13℃ to 29.68℃ temperature range. No significant effects were observed with DNI or the antagonist protocol. These findings suggest that summer treatment may optimize IVF outcomes, but future studies should prioritize multi-center prospective designs with continuous temperature monitoring to define precise optimal ranges.

**Supplementary Information:**

The online version contains supplementary material available at 10.1007/s00484-025-02951-2.

## Background

The role of seasonal variations in conception in mammals has a long history of investigation (Đuričić et al. 2019; Ingraham et al. 1974; Steinbach and Balogun 1971). Many studies have reported seasonal variations in natural conception in humans (Martinez-Bakker et al. 2014; Wesselink et al. 2020). It has been posited that natural conception is influenced by environmental factors, including ambient temperature and light exposure. Evidence suggests that men’s sperm quality is related to temperature fluctuations, as both low and high ambient temperatures are significantly associated with decreased semen quality (Zhou et al. 2020). A study found that exposure to higher ambient temperatures is associated with reduced ovarian reserve (Gaskins et al. 2021). Additionally, extreme heat can impair DNA repair mechanisms, affecting pre-implantation embryo development (Qu et al. 2024), while cold exposure may lead to female reproductive dysfunction through the ovarian PERK/NRF2/CX43/StAR/progesterone pathway (Ding et al. 2024). These findings highlight the importance of avoiding extreme temperatures when planning for conception or undergoing fertility treatments. The above evidence suggests ambient temperature may influence gametogenesis through distinct mechanisms. Moreover, light exposure has been shown to influence natural conception. A study found that exposure to bright light in the morning can stimulate the secretion of reproductive hormones, promote follicular growth in the ovaries, and increase the ovulation rate (Danilenko and Samoilova 2007). Another study found that women aged 30–40 are more likely to conceive in the summer, possibly due to the positive effects of solar radiation on ovarian function (Parikh et al. 2023). These findings suggest that optimizing light exposure and timing fertility treatments during favorable seasons could improve conception rates. Therefore, it can be inferred that meteorological factors play a significant role in reproductive health. Although environmental factors increasingly affect natural conception, their impact on IVF success remains uncertain. Major questions persist about whether ovarian stimulation protocols help mitigate external stressors, or if environmental exposures during crucial IVF phases - particularly folliculogenesis - meaningfully affect treatment success. Furthermore, when spontaneous pregnancies are evaluated, the influence of meteorological changes on human reproduction may be obscured by various other factors, including sociocultural influences, sexual activity patterns, and additional related variables.

Assisted reproductive technology serves as an exemplary model for examining the impact of meteorological variations on reproduction during periconceptional periods, given that both the physiological status of patients and meteorological variables can be accurately and precisely assessed. Previous studies on the impact of season on IVF outcomes have yielded conflicting results. For example, a study in Liverpool found that implantation and pregnancy rates were significantly higher in summer cycles compared to other seasons (Wood et al. 2006). In contrast, a study in Bristol reported no significant differences in pregnancy rates across various months of the year (Fleming et al. 1994). Similarly, a recent study in Zhengzhou, China, observed elevated clinical pregnancy rates in ovulation induction cycles that began during spring, summer, and autumn, compared to those initiated in winter (Chu et al. 2022). However, these findings were contradicted by research conducted in Turkey, which demonstrated that the month or season of fresh and frozen embryo transfer had no significant impact on clinical pregnancy rates (Korkmaz et al. 2023). In summary, the existing research on the impact of season on IVF outcomes presents inconsistent findings. While some studies suggest potential benefits of certain seasons, others find no significant effect. These inconsistencies may be attributed to differences in study populations, criteria for assigning patients to specific seasons (e.g., based on stimulation day, oocyte retrieval day, or embryo transfer day), methods of estimating meteorological changes (only averaging the values over a month or a season), and geographical locations. Therefore, it is crucial to interpret these results with caution and consider additional research to clarify the relationship between meteorological factors and IVF success. Notably, the ovarian stimulation to oocyte retrieval phase represents a biologically critical window during which meteorological factors may directly affect follicular development and oocyte quality, thereby influencing IVF outcomes. However, prior analyses often aggregated data across broad seasons or months, potentially diluting subtle effects specific to this sensitive period.

Thus, given existing literature’s inconsistencies and controversies on environmental impacts on IVF pregnancy outcomes, we conducted a retrospective cohort study to assess the effects of season, temperature, and DNI on IVF pregnancy outcomes. This study investigates the relationship between meteorological conditions during the ovarian stimulation-to-oocyte retrieval phase and pregnancy outcomes, aiming to provide more precise insights into how environmental factors might influence IVF success. This targeted strategy based on environmental factors can offer practical guidance for optimizing IVF treatment protocols.

## Materials and methods

### Study design and population

This retrospective cohort study was conducted at the Reproductive Hospital of Guangxi Zhuang Autonomous Region, a tertiary-care facility specializing in assisted reproductive technology. We used data from first-time IVF patients who underwent oocyte retrieval between June 2021 and October 2023. The hospital is situated in Nanning, Guangxi, China, a region characterized by a humid subtropical climate with marked seasonal variations based on the Köppen-Geiger classification system (Peel et al. 2007). The inclusion criteria were as follows: (1) age ≤ 45 years at cycle initiation, to minimize age-related confounding effects on ovarian response and embryo quality; (2) first-time fresh IVF cycle using a long protocol or antagonist protocol, excluding prior cycles confounding or alternative protocols to standardize ovarian stimulation regimens and ensure treatment consistency; and (3) transfer of fresh embryos. Only stimulation followed by fresh embryo transfer accurately captures the constant effect of meteorological variations. The exclusion criteria were as follows: (1) preimplantation genetic testing cycles, as preimplantation genetic testing introduces embryo selection bias and additional laboratory interventions (e.g., biopsy, extended culture) that may independently affect outcomes; and (2) embryo transfer in a frozen cycle, to eliminate variability from cryopreservation protocols; and (3) cycles with incomplete data, ensuring robustness of the dataset for analysis.

### Stimulation protocol

In this study, the stimulation protocols used were the long protocol and the antagonist protocol. The long protocol‌ initiates with GnRH agonist administration to suppress gonadotropin-releasing hormone (GnRH), downregulating pituitary activity and gonadotropin production. This preparation phase is followed by ovarian stimulation with exogenous gonadotropins to promote follicle development and oocyte maturation‌. The long protocol is notable for its extended duration, often lasting several weeks, and its focus on comprehensive pituitary suppression before ovarian stimulation. ‌‌The antagonist protocol‌ uses a GnRH antagonist to control ovarian stimulation by preventing premature ovulation. The antagonist binds to the GnRH receptor, inhibiting the pituitary’s response to endogenous GnRH and allowing for precise control of ovarian stimulation. The duration of ovarian stimulation with the antagonist protocol is generally shorter compared to the long protocol.

### Seasonal and meteorological parameters

**Fig. 1:**
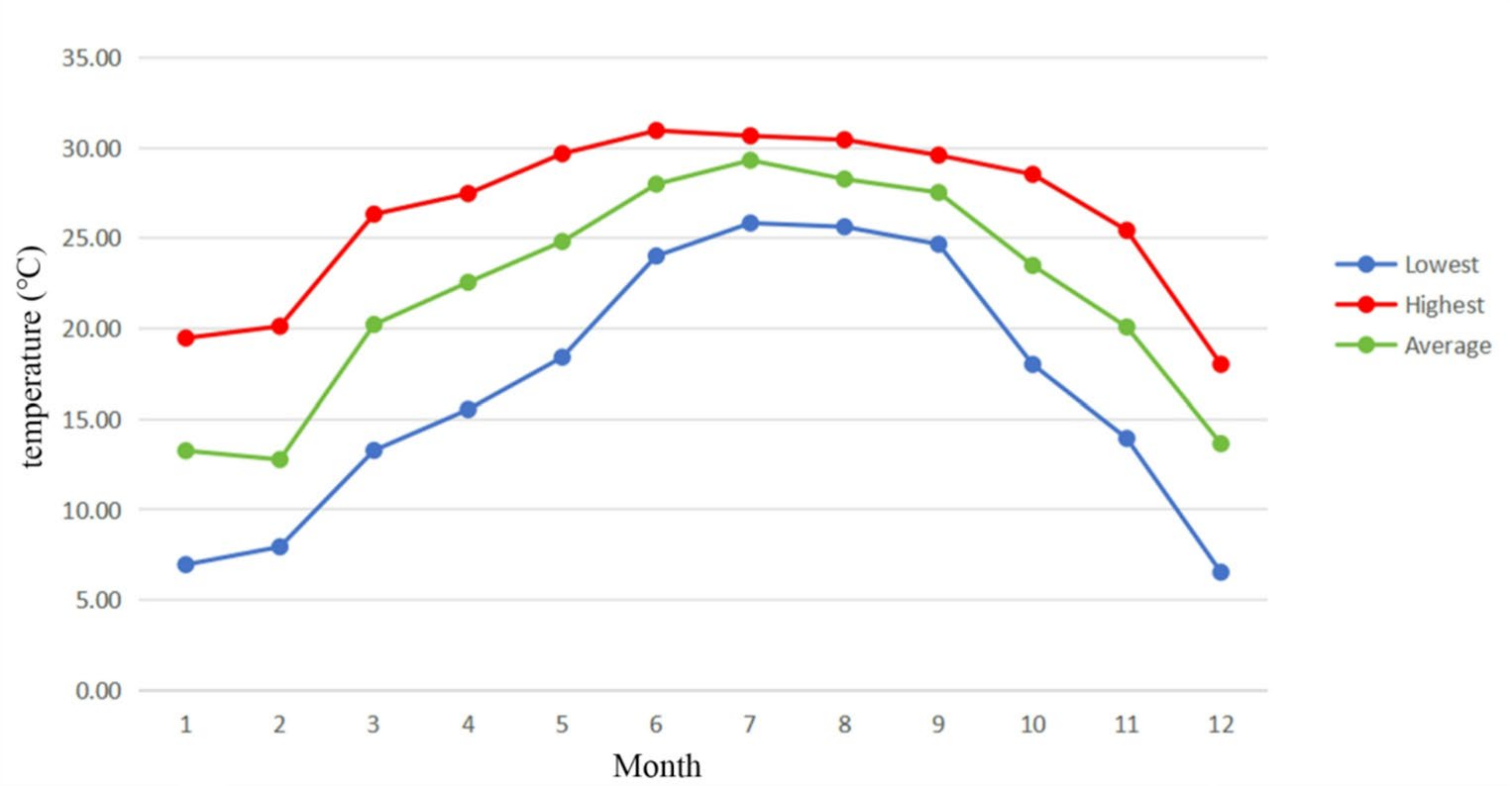
Displays monthly temperature combined June 2021 to October 2023. The green line represents the average temperature, the red line indicates the average highest temperature, and the blue line shows the average lowest temperature. Each data point represents the temperature value calculated as the average of the corresponding monthly temperatures over the entire period from June 2021 to October 2023

Figure 1 shows the monthly temperature data combined from June 2021 to October 2023, indicating that the seasonal characteristics of Nanning city are obvious. Figure S1 shows nanning meteorological data source areas and participant recruitment sites. Patients were divided into four groups (spring, summer, autumn, and winter) based on the date of oocyte retrieval. The seasons were defined based on the calendar for Guangxi before data analysis. Each season lasts 3 months, as follows: spring (March 1 to May 31), summer (June 1 to August 31), autumn (September 1 to November 30), and winter (December 1 to February 28/29). The meteorological data for this study, spanning from June 2021 to October 2023, were collected for Nanning city, Guangxi Zhuang Autonomous Region, China. Specifically, the data included hourly temperature and direct normal irradiance (DNI) measurements. The definition of DNI as the direct irradiance received on a plane normal to the sun over the total solar spectrum (Blanc et al. 2014). These environmental parameters were chosen because temperature variations can influence the physiological processes of both patients and developing oocytes (LaPointe et al. 2025), while DNI is a key indicator of solar radiation intensity, which may influence patients’ exposure to sunlight and subsequent biological effects (Parikh et al. 2023). Meteorological data were collected daily during the treatment period, spanning from the initiation of Gn administration to the day of oocyte retrieval. The overall average temperature and average DNI during the treatment period, from the initiation of Gn administration to the day of oocyte retrieval, were calculated and analyzed. The data utilized in this study were obtained from the ERA5 historical reanalysis dataset of the European Centre for Medium-Range Weather Forecasts (ECMWF), and is accessible via the Xihe Energy Big Data Platform (https://xihe-energy.com/). (Hersbach et al. 2018) ERA5 was designed as the successor to the ERA-Interim reanalysis (Dee et al. 2011) offering substantial advancements including enhanced spatial resolution (0.25°×0.25°), hourly temporal resolution, and expanded vertical coverage. This dataset, spanning from 1959 onward, integrates a sophisticated 12-hourly 4DVar data assimilation framework with multi-source inputs such as numerical model outputs, historical satellite retrievals, and ground-based observational records. The Xihe Energy Big Data Platform further ensures rigorous data quality control during collection, processing, and analysis, guaranteeing completeness and validity.

### Embryological and pregnancy outcome measures

The main outcome measure was clinical pregnancy. Clinical pregnancy was defined as one or more gestational sacs confirmed by ultrasound. Clinical pregnancy serves as a crucial endpoint in IVF studies, providing a definitive indication of the efficacy of the IVF protocol. The secondary outcome measures included the number of oocytes retrieved, the number of mature oocytes, the number of 2 pronuclei (PN), and the number of available embryos. These secondary outcomes are pivotal in determining IVF pregnancy outcomes and may influence pregnancy outcomes.

### Ethics approval and consent to participate

The study has been performed in accordance with the Declaration of Helsinki, and received approval from the Institutional Review Board at Reproductive Hospital of Guangxi Zhuang Autonomous Region (ethics approval number:KY-LL-2023-009). All patients had given consent for their data to be used for analysis. To ensure participant privacy, we removed all patients' names, phone numbers, and other direct personal identifiers (such as ID numbers) from the research data. Each study participant was assigned a unique study ID, which served as the sole identifier for all data recording and analysis. Furthermore, access to the electronic data was restricted to authorized personnel only.

### Statistical analysis

Median and interquartile range (IQR) were used to describe continuous variables, and proportion (%) was used to describe categorical variables. The Kruskal‒Wallis test and chi‒square test were used to compare the variables across different seasons. Binary logistic regression analysis was performed to assess the association between season and clinical pregnancy (a binary outcome categorized as successful or not). This method was chosen because it is specifically designed for analyzing relationships between categorical outcomes and multiple predictors, providing interpretable odds ratios while accommodating both categorical and continuous covariates (Wang et al. 2019). Model fit was assessed using the Hosmer-Lemeshow goodness-of-fit test (*p* > 0.05 indicating adequate calibration) and variance inflation factors (VIFs < 5 for all covariates) to check for multicollinearity. The RCS regression was performed to analyze the dose‒response relationship between average temperature, average DNI and clinical pregnancy. This approach was selected for its ability to model complex non-linear relationships while maintaining parsimony through restricted flexibility between knots (Desquilbet and Mariotti 2010). The number of knots (k = 4) was determined via Akaike Information Criterion (AIC)-based optimization from 3 to 5 candidate knots, balancing curve smoothness against overfitting risks. Nonlinearity was formally tested using likelihood ratio tests comparing linear and spline models (*p* < 0.05 for nonlinear terms), with multicollinearity again assessed via VIFs (< 5 for all terms). Missing data occurred in 54 cases (4.38% of the sample). Little’s Missing Completely at Random (MCAR) test yielded (χ² = 29.614, df = 23, *p* = 0.161), supporting the validity of listwise deletion. This supported the appropriateness of listwise deletion for handling missing values in subsequent analyses. We also conducted stratified analysis by protocol (long protocol and antagonist protocol) to evaluate the robustness of the results. To control for potential confounders in the analysis, covariates were carefully selected based on previous literature and univariate analysis results. Covariates were included in the adjusted regression models if they met either of the following criteria: (1) they were previously reported to be associated with clinical pregnancy (Mozurkewich 2020; Huang et al. 2024; He et al. 2022; van Rumste et al. 2000); or (2) they exhibited a P value < 0.1 in univariate analysis (Table S1). The selected covariates for inclusion in both the binary logistic regression and RCS regression models were age, race, occupation, infertility duration, type of infertility, infertility factor, protocol type (excluding models stratified by protocol), Gn dosage, intimal thickness at trigger, fertilization method, day of transfer, and follicle-stimulating hormone (FSH) baseline. All the data were analyzed using SPSS 22.0 software and R 4.0.3. The 95% confidence interval (95%CI) not containing a value of 1 and *P* < 0.05 (two-tailed) were regarded as statistically significant.

## Results

### Patient general characteristics and laboratory and pregnancy outcomes

Table 1 shows the demographic and clinical characteristics of the patients. A total of 1179 patients (1179 fresh IVF cycles) satisfied the inclusion and exclusion criteria. Patients were divided into four groups based on the date of oocyte retrieval: 229 patients in the spring group, 422 patients in the summer group, 392 patients in the autumn group, and 136 patients in the winter group. Age, race, occupation, type of infertility, infertility factor, FSH baseline, Gn dosage, intimal thickness at trigger, day of transfer, number of mature oocytes, number of 2PN fertilizations, and number of available embryos were not significantly different among seasons (*P* > 0.050). The duration of infertility (*P* = 0.008), protocol (*P* = 0.040), fertilization method (*P* = 0.023), and number of oocytes retrieved (*P* = 0.028) differed among seasons.

**Table 1:** Demographic and clinical characteristics by season at the time of oocyte retrieval

Characteristics	Spring	Summer	Autumn	Winter	*P*
No. of ET cycles(n)	229	422	392	136	
Age (years)	36 (32–39)	35 (32–38)	35 (32–39)	36 (33–39)	0.265
Race					0.979
Han	110 (48.03)	199 (47.16)	191 (48.73)	62 (45.59)	
Zhuang	107 (46.73)	205 (48.58)	183 (46.68)	66 (48.53)	
Others	12 (5.24)	18 (4.26)	18 (4.59)	8 (5.88)	
Occupation					0.515
Employed	104 (45.42)	180 (42.65)	182 (46.43)	60 (44.12)	
Agriculturial	21 (9.17)	28 (6.64)	24 (6.12)	6 (4.41)	
Others	104 (45.41)	214 (50.71)	186 (47.45)	70 (51.47)	
Duration of infertility (years)	3.50 (2.00–6.00)	4.00 (2.00–6.00)	3.00 (2.00–5.00)	3.00 (1.73–5.00)	0.008
Type of infertility					0.172
Primary	106 (46.29)	159 (37.68)	166 (42.35)	59 (43.38)	
Secondary	123 (53.71)	263 (62.32)	226 (57.65)	77 (56.62)	
Infertility factor					0.224
Tubal factor	36 (15.72)	81 (19.19)	72 (18.37)	28 (20.59)	
Uterine factor	1 (0.44)	1 (0.24)	5 (1.27)	0 (0.00)	
Female mixed factor	69 (30.13)	132 (31.28)	109 (27.81)	39 (28.68)	
Male mixed factor	10 (4.37)	16 (3.79)	29 (7.40)	6 (4.41)	
Both female and male factor	108 (47.16)	191 (45.26)	170 (43.37)	61 (44.85)	
Diminished ovarian reserve	3 (1.31)	0 (0.00)	2 (0.51)	0 (0.00)	
Unexplained	2 (0.87)	1 (0.24)	5 (1.27)	2 (1.47)	
FSH baseline (IU/L)	7.47 (6.40–8.86)	7.17 (6.13–8.73)	7.41 (6.14–8.9)	7.26 (6.20–8.77)	0.663
Protocol					0.040
Long	100 (43.67)	196 (46.45)	202 (51.53)	52 (38.24)	
Antagonist	129 (56.33)	226 (53.55)	190 (48.47)	84 (61.76)	
Gn dosage (IU)	2400.00 (1800.00–3000.00)	2475.00 (1875.00–3225.00)	2550.00 (1846.88–3450.00)	2412.50 (1800.00–3225.00)	0.379
Intimal thickness at trigger (mm)	10.00 (8.80–11.30)	10.00 (8.80–11.40)	10.20 (9.00–11.78)	10.00 (8.50–11.40)	0.325
Fertilization method					0.023
IVF	185 (80.79)	351 (83.18)	307 (78.32)	122 (89.71)	
ICSI	44 (19.21)	71 (16.82)	85 (21.68)	14 (10.29)	
Day of transfer					0.051
D3	154 (67.25)	256 (60.66)	264 (67.35)	98 (72.06)	
D5	75(32.75)	166 (39.34)	128 (32.65)	38 (27.94)	
Number of oocytes retrieved	9.00 (5.50–12.00)	9.00 (6.00–13.00)	9.00 (6.00–13.00)	8.00 (5.00–11.00)	0.028
Number of mature oocytes	7.00 (4.00–10.00)	8.00 5.00–11.00)	7.00 (5.00–10.75)	6.00 (4.25–10.00)	0.086
Number of 2PN fertilizations	5.00 (3.00–7.00)	5.00 (3.00–8.00)	5.00 (3.00–7.75)	5.00 (3.00–7.00)	0.116
Number of available embryos	3 (2–5)	3 (2–4)	3 (2–4)	3 (2–4)	0.068

Data are presented as median (interquartile range) for quantitative variables and n (%) for categorial variables

*ET* embryo transfer; *FSH* follicle-stimulating hormone; *Gn* gonadotropin; *IVF* in vitro fertilization; *ICSI* Intracytoplasmic Sperm Injection; *PN* pronuclei

### Seasonal patterns in pregnancy outcomes

We used binary logistic regression analysis to assess the association between season and clinical pregnancy. The binary logistic regression model demonstrated a good fit (Hosmer-Lemeshow test, χ²=5.32, *p* = 0.723) with no significant multicollinearity concerns (all VIFs < 2), supporting the reliability of the model for statistical inference. Season has a significant association with pregnancy probability. Compared to those in winter, patients had a 1.74 times higher likelihood of pregnancy in spring (95% CI:1.11–2.71, *P* = 0.015) and a 1.53 times higher likelihood in summer (95% CI:1.02–2.30, *P* = 0.042), respectively. Moreover, we conducted stratified analysis by protocol (long protocol and antagonist protocol) to evaluate the correlation between season and clinical pregnancy. For the long protocol, compared to those in winter, patients had a 2.02 times higher likelihood in summer (95% CI:1.07–3.82, *P* = 0.031). However, for the antagonist protocol, the probability of clinical pregnancy in the other three seasons was similar to that observed in winter (Table 2). This highlights that the likelihood of pregnancy was higher in spring and summer compared to winter, particularly with the long protocol during summer. In contrast, the antagonist protocol showed no significant seasonal variation in pregnancy probability.

**Table 2:** Logistic regression analysis of the effect of seasonal variables on the clinical pregnancy

season	All(*n* = 1179 cycles)			Long protocol(*n* = 550 cycles)			Antagonist protocol(*n* = 629 cycles)		
	OR	95% CI	*P*	OR	95% CI	*P*	OR	95% CI	*P*
Winter	1	(Reference)		1	(Reference)		1	(Reference)	
Spring	1.74	1.11, 2.71	0.015	1.46	0.73, 2.93	0.285	1.76	0.98, 3.15	0.059
Summer	1.53	1.02, 2.30	0.042	2.02	1.07, 3.82	0.031	1.16	0.68, 1.98	0.600
Autumn	1.12	0.74, 1.69	0.597	0.98	0.52, 1.84	0.938	1.24	0.72, 2.16	0.436

*OR* odds ratio

Adjusted for age, race, occupation, infertility duration, type of infertility, infertility factor, protocol (except in models stratified by protocol), Gn dosage, intimal thickness at trigger, fertilization method, day of transfer, follicle-stimulating hormone (FSH) baseline

### Effects of meteorological changes on pregnancy outcomes

**Fig. 2:**
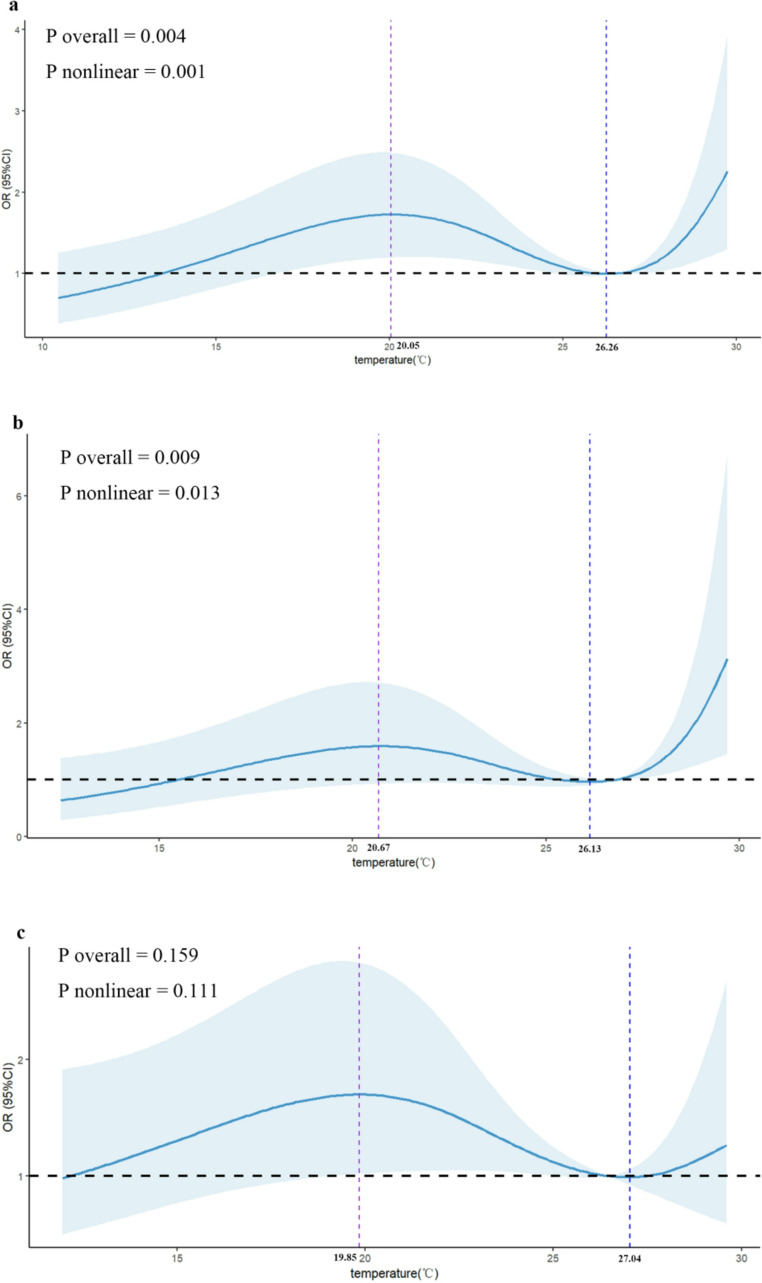
Depicts the association between clinical pregnancy odds and average temperature during IVF cycles, modelled using restricted cubic splines (RCS). The model adjusts for key covariates such as age, race, occupation, infertility duration, type of infertility, infertility factor, protocol (except in models stratified by protocol), Gn dosage, intimal thickness at trigger, fertilization method, day of transfer, follicle-stimulating hormone (FSH) baseline. Panel (**a**) represents all patients, panel (**b**) focuses on the long protocol subgroup, and panel (**c**) includes the antagonist protocol subgroup. Shaded areas represent 95% CI

**Fig. 3:**
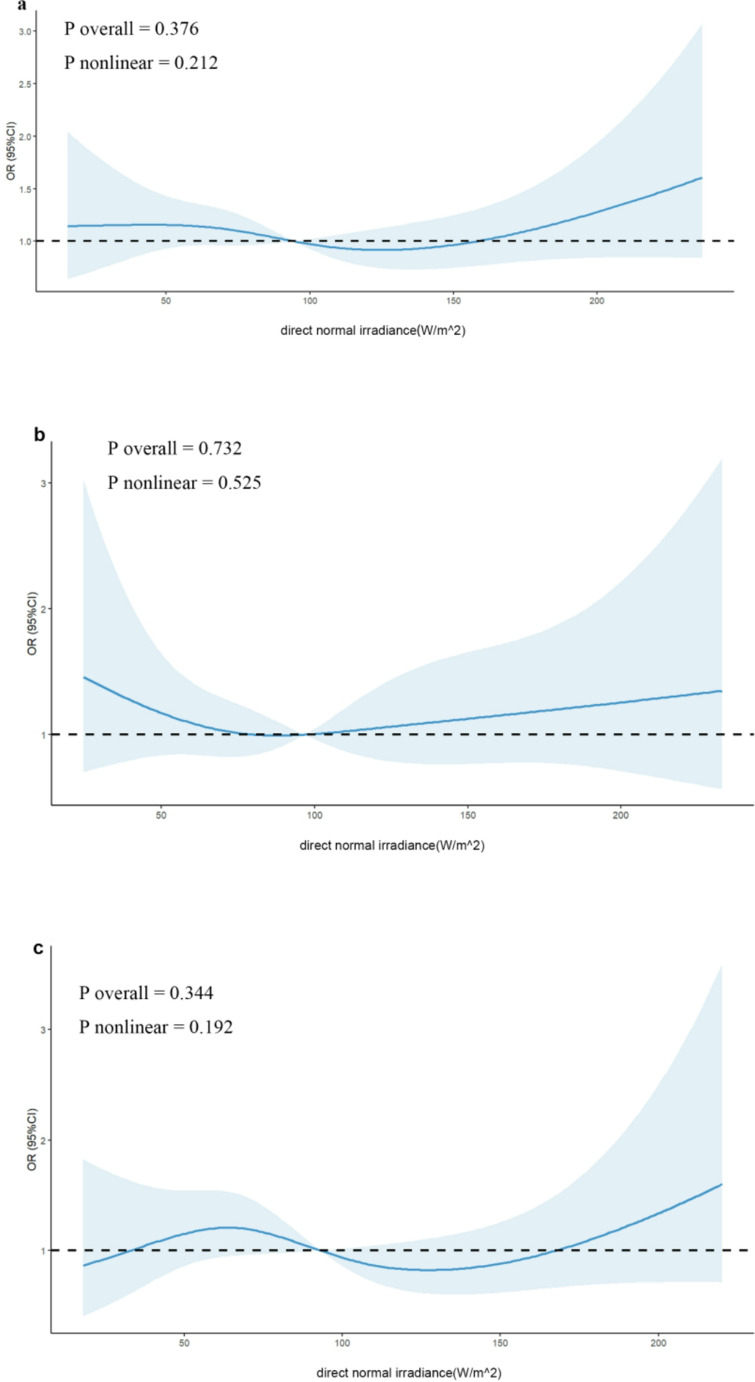
Depicts the association between clinical pregnancy odds and average direct normal irradiance during IVF cycles, modelled using restricted cubic splines (RCS). The model adjusts for key covariates such as age, race, occupation, infertility duration, type of infertility, infertility factor, protocol (except in models stratified by protocol), Gn dosage, intimal thickness at trigger, fertilization method, day of transfer, follicle-stimulating hormone (FSH) baseline. Panel (**a**) represents all patients, panel (**b**) focuses on the long protocol subgroup, and panel (**c**) includes the antagonist protocol subgroup. Shaded areas represent 95% CI. Despite adjusting for key covariates, there were no significant overall or nonlinear dose-response correlations between average direct normal irradiance and clinical pregnancy. The absence of significant findings may suggest that direct normal irradiance is not a major determinant of clinical pregnancy outcomes in the context of IVF cycles, or that the effect, if any, is too subtle to be detected within the study’s sample size and statistical power, and a larger sample could potentially reveal a clearer association. Additionally, high individual variability in irradiance susceptibility and unaccounted confounding factors, such as lifestyle, genetics, and health conditions, could have influenced the results and masked any significant trends

We used RCS regression to analyze the correlation between average temperature and average DNI and clinical pregnancy. ‌The optimal knot placement for the restricted cubic spline analysis was determined via AIC comparisons, with 4 knots (5 th, 35 th, 65 th, and 95 th percentiles) demonstrating superior fit (AIC = 1556.93) compared to both linear (linear AIC = 1565.13, ΔAIC = 8.20) and alternative spline configurations (knots = 3: AIC = 1566.00, ΔAIC = 9.07; knots = 5: AIC = 1555.89, ΔAIC = 1.04). The nonlinear model was statistically confirmed through likelihood ratio test (χ²=16.202, df = 4, *p* = 0.003), and model diagnostics revealed no multicollinearity concerns (all variance inflation factors < 2). The RCS regression analysis revealed a nonlinear association between average temperature and clinical pregnancy outcomes, indicating significant temperature variations that influence the likelihood of pregnancy (P overall = 0.004, P nonlinear = 0.001). Specifically, for an average temperature of ≤ 20.05℃, there was a moderate elevation in the odds of clinical pregnancy, suggesting that patients undergoing IVF may benefit from treatments conducted in this temperature range. Conversely, as the average temperature ranged between 20.05℃ and 26.26℃, there was a moderate decrease in the odds of clinical pregnancy, highlighting the need for clinicians to be cautious when scheduling treatments during this period. Notably, the odds of clinical pregnancy experienced a sharper increase when the average temperature ranged between 26.26℃ and 29.75℃, which may prompt further investigation into the optimal temperature range for IVF treatments. (Fig. 2a). There were no significant overall or nonlinear dose‒response correlations between average DNI and clinical pregnancy (P overall = 0.376, P nonlinear = 0.212) (Fig. 3a). Furthermore, we conducted stratified analysis by protocol (long protocol and antagonist protocol) to evaluate the correlation between average temperature and average DNI and clinical pregnancy. For the long protocol, a nonlinear correlation between average temperature and clinical pregnancy was observed (P overall = 0.009, P nonlinear = 0.013). Specifically, there was a moderate increase in the likelihood of clinical pregnancy when the average temperature was ≤ 20.67℃. However, as the temperature rose to between 20.67℃ and 26.13℃, there was a moderate decrease in the odds of clinical pregnancy. Notably, a sharper increase in the odds of clinical pregnancy was observed when the temperature ranged from 26.13℃ to 29.68℃ (Fig. 2b). For the antagonist protocol, no significant overall or nonlinear dose‒response correlations were observed between average temperature and clinical pregnancy (P overall = 0.159, P nonlinear = 0.111) (Fig. 2c). This difference between protocols suggests that the impact of temperature on IVF outcomes may be protocol-specific, further emphasizing the need for tailored treatment plans based on individual patient characteristics and protocols. There were no significant overall or nonlinear dose‒response correlations between average DNI and clinical pregnancy in either the long protocol (P overall = 0.732, P nonlinear = 0.525) (Fig. 3b) or the antagonist protocol (P overall = 0.344, P nonlinear = 0.192) (Fig. 3c). This highlights that while temperature variations play a significant role, other environmental factors such as DNI do not appear to influence IVF outcomes significantly in our study.

## Discussion

In the present study, we found that season and environmental temperature significantly altered clinical pregnancy. Among all patients, the odds of clinical pregnancy were significantly greater in spring and summer than in winter. Furthermore, we conducted stratified analysis by protocol. For the long protocol, the odds of clinical pregnancy in the summer were greater than those in the winter. In contrast, no significant associations were detected in the antagonist protocol. Moreover, a nonlinear association between average temperature and clinical pregnancy was confirmed in all patients and the long protocol subgroup. However, this association was not observed in the antagonist protocol subgroup. Additionally, in all patients and in both the long protocol and the antagonist protocol subgroups, no significant overall or nonlinear dose‒response correlations were observed between the average DNI and clinical pregnancy. These findings suggest that season and temperature play a significant role in influencing the success rates of clinical pregnancies. It is advisable for IVF treatment protocols to consider season and temperature ranges to optimize outcomes.

There is much controversy as to whether season and meteorological factors are related to pregnancy outcomes during IVF treatment. For instance, a study in Zhengzhou City, Henan Province, China reported that the clinical pregnancy rate of ovarian stimulation cycles started in spring, summer and autumn was significantly higher than that of ovarian stimulation cycles started in winter (Chu et al. 2022). Similarly, a study with 2709 cycles in Liverpool demonstrated that pregnancy rate in summer (April–September) was better than that in winter (October–March) (Wood et al. 2006). However, conflicting findings, such as increased pregnancy rates from November to February in the Netherlands (Stolwijk et al. 1994), underscore the complexity of this relationship. These discrepancies may stem from stimulation protocol, methodological variations, geographic variability in environmental factors, differences in study populations.

Our stratified analysis by protocol revealed significantly higher clinical pregnancy odds in summer than winter for the long protocol, aligning with Zhengzhou (Chu et al. 2022) and Liverpool (Wood et al. 2006) studies, which also used the long protocol. Conversely, no seasonal effect was observed for the antagonist protocol, consistent with findings from Hong Kong (Zhao et al. 2019). The long protocol and antagonist protocol differ in physiological mechanisms. The long protocol typically involves prolonged GnRH agonist administration, which may render it more sensitive to environmental factors such as temperature fluctuations. In contrast, the antagonist protocol, characterized by shorter medication duration and rapid hormonal suppression, likely exhibits lower sensitivity to ambient temperature variations (Abulajiang et al. 2023). Furthermore, the extended follicular development period in the long protocol may heighten susceptibility to temperature changes, while the antagonist protocol’s rapid follicular development process may make it more adaptive to temperature changes (Liu et al. 2012). This suggests that stimulation protocols modulate seasonal effects, possibly through interactions with hormonal responses or follicular development under varying climatic conditions. However, the exact mechanisms remain unclear and warrant further investigation.

Methodological differences likely contribute to inconsistent conclusions. Studies measuring temperature at different treatment phases (e.g., ovulation induction vs. oocyte retrieval) reported opposing associations. For example, a study conducted in Zhengzhou reported that an elevated daily average temperature at the time of ovulation induction was associated with an increased clinical pregnancy rate (Chu et al. 2022). However, a study conducted in Taiwan found that temperature at the time of oocyte retrieval was inversely associated with the pregnancy rate (Chang et al. 2005). Our study relied on meteorological averages obtained during the pivotal treatment phase, spanning from the initiation of Gn administration to the day of oocyte retrieval. This approach provided higher accuracy in measuring average temperature compared to the studies in Zhengzhou and Taiwan. Additionally, as shown in Figs. 2, our study used the RCS regression model, revealed nonlinear temperature effects, indicating thresholds beyond which temperature impacts diminish or reverse. This contrasts with studies using multivariate logistic regression model model (Chu et al. 2022) or Pearson correlation analysis model (Chang et al. 2005), which lack capacity to capture such complexity. Furthermore, our findings, which showed no significant association between DNI and IVF outcomes, contrast with the Taiwanese study demonstrating negative correlations between cumulative light hours and reproductive success rates (Chang et al. 2005). This discrepancy likely arises from fundamental differences in our measurement parameters. While both studies examined light-related variables, DNI quantifies solar radiation intensity whereas cumulative light hours measures cumulative exposure time, representing distinct biological mechanisms that may differentially influence reproductive physiology.

Geographic and climatic heterogeneity also plays a critical role. Our study found higher clinical pregnancy odds in summer than winter, conflicting with findings in the Netherlands (Stolwijk et al. 1994). Nanning’s subtropical climate (warm, distinct seasons) contrasts sharply with the Netherlands’ temperate maritime climate (mild summers, cooler winters). Such differences may affect seasonal patterns of vitamin D synthesis, melatonin secretion, or pollutant exposure, all implicated in reproductive outcomes. Moreover, population-specific factors, including ethnic diversity, baseline health, and lifestyle habits (e.g., diet, physical activity), could interact with environmental variables, amplifying or attenuating seasonal effects.

The mechanisms underlying the impacts of season and temperature on clinical pregnancy are not fully understood. Compared with summer, vitamin D deficiency is more common in winter (Andersen et al. 2013; Kroll et al. 2015; Schramm et al. 2017). Previous research have implied that season may affect IVF outcomes through serum vitamin D levels, which tend to increase in the summer months (Farland et al. 2020; Vandekerckhove et al. 2016). A systematic review of 11 cohort studies found that sufficient vitamin D levels were associated with higher clinical pregnancy and live birth rates in women undergoing ART (Chu et al. 2018). However, a recent systematic review and meta-analysis by Cozzolino et al. suggest that vitamin D may have limited influence on clinical pregnancy rates, contrasting with earlier studies (Cozzolino et al. 2020). This highlights the need for further research to clarify the role of vitamin D in ART outcomes and to better understand the potential mechanisms involved. Additionally, the impact of temperature on pregnancy outcomes remains an area of active research. There is significant scientific uncertainty regarding the specific effects of heat/cold on pregnancy outcomes, and the underlying physiological mechanisms are not yet fully understood (Bonell et al. 2024). Wellcome Trust has funded a series of studies on biological pregnancy and heat, including one conducted in Gambia. The results from Gambia indicate that reducing maternal exposure to heat stress and heat strain is likely to alleviate fetal strain, potentially leading to a reduction in adverse birth outcomes (Bonell et al. 2022). However, in a cross-sectional study conducted in Ghana, researchers found no significant association between heat exposure and miscarriage or stillbirth, suggesting that the impact of heat exposure on pregnancy outcomes may be limited in certain regions (Asamoah et al. 2018). Our findings indicate that temperature, rather than DNI, is the primary factor driving the association between season and clinical pregnancy. Thus, this study highlights that temperature may be an independent regulator of clinical pregnancy outcomes, but the physiological mechanisms involved need to be further studied.

To our knowledge, ovarian function is associated with IVF outcomes (Cakiroglu et al. 2022; Correia et al. 2022; Gleicher et al. 2007). A previous study demonstrated that prepubertal exposure to high temperature impairs ovarian function by diminishing the expression of steroidogenic enzymes, estrogen and gonadotropin receptors in the ovaries of female rats (Zheng et al. 2019). Another study found a negative association between ambient temperature and the antral follicle count (AFC) in patients with infertility (Gaskins et al. 2021). However, our results revealed that the number of oocytes retrieved in winter was lower than that in other seasons, which was inconsistent with the higher AFC at cooler temperatures. Nevertheless, the AFC, as an indicator of ovarian reserve function, does not necessarily represent oocyte quality. Higher ambient temperature may be related to the improvement in oocyte quality. Surprisingly, our study found that the number of mature oocytes was highest in summer and lowest in winter. Thus, we suspect that higher ambient temperatures may be positively influence oocyte maturation and quality. The potential mechanisms linking ambient temperature to clinical pregnancy outcomes in IVF may involve multifaceted hormonal regulation, particularly through the hypothalamic-pituitary-gonadal (HPG) axis and ovarian steroidogenesis. Chronic cold stress in rodents suppresses hypothalamic kisspeptin expression, leading to attenuated GnRH release and disrupted LH surges (Retana-Márquez et al. 2020). Based on findings from rodent studies, we hypothesize that humans may possess a similar mechanism, which could partially account for the observed seasonal decline in oocyte retrieval during winter months. However, whether this mechanism applies to humans requires further investigation. In addition, it is possible that lifestyle changes associated with activities and diet during the warmer months be contributing to better clinical outcomes. Air pollution may be another factor, and lower air pollution in the summer months may be responsible for better clinical outcomes (Cichowicz et al. 2017). Therefore, future researches should take into account the aforementioned factors that may be influenced by season to the greatest extent possible. It could be beneficial to schedule more IVF treatments during the warmer months, especially in regions with distinct seasonal temperature variations. Clinics could consider scheduling IVF cycles during warmer seasons (such as summer) to optimize outcomes. However, it is important to note that other factors such as patient age, and individual health conditions should also be considered when making such decisions.

It is worth noting that this study has several advantages. First, we took into account only the first fresh cycle for each patient, thereby removing the dependency between cycles from the same patient. Second, our study relied on meteorological averages obtained during the pivotal treatment phase, spanning from the initiation of Gn administration to the day of oocyte retrieval. This approach allowed us to more precisely study the influence of meteorological changes on clinical outcomes. Third, our study employed RCS to model nonlinear relationships between temperature and IVF outcomes, providing insights into how temperature thresholds and inflection points affect success rates. Finally, the stratified analysis we conducted by protocol type (long versus antagonist) revealed significant associations in the long protocol group, which further deepened and enriched the findings.

Nevertheless, there are also inevitable limitations to our study. First, the study focused solely on first-time fresh IVF cycles, excluding frozen and subsequent cycles. This exclusion overlooks cumulative success rates, which are often more relevant to patients. Future studies could expand the scope to include patients through subsequent frozen and repeated cycles, providing a more comprehensive understanding of the long-term effects of temperature and other meteorological factors on IVF outcomes. Second, the data for this study were obtained from a single institution. The single-center design in a subtropical region limits extrapolation to populations in contrasting climates (e.g., arid, temperate, or polar zones). Future studies should explore the impact of these environmental factors across multiple geographic locations with varied climates to enhance the generalizability of these findings. Third, the impact of meteorological conditions prior to the initiation of IVF cycles (e.g., 3–6 months prior to ovarian stimulation) was not evaluated in this study. Future studies could integrate extended environmental monitoring to capture pre-conception exposures and their lagged effects. Finally, it is important to acknowledge that this study did not incorporate socio-cultural confounders such as lifestyle, diet, or psychological stress, all of which could intersect with environmental factors to impact IVF outcomes. Future research should aim to collect data on these variables to enhance the reliability of the study results.

## Conclusions

In conclusion, our study demonstrates that season and temperature significantly impact IVF pregnancy outcomes, particularly in the long protocol. IVF pregnancy rates are higher in spring and summer, especially in summer for the long protocol, compared to winter. Temperature and pregnancy likelihood have a nonlinear link, with the most notable rise between 26.13℃ and 29.68℃ in the long protocol. No significant effects were observed for DNI or the antagonist protocol. Based on our findings, scheduling IVF treatments during warmer seasons (particularly in summer) or under ambient temperatures approximating 26–30 °C may improve pregnancy outcomes for patients undergoing the long protocol, though further validation is required. Future studies should prioritize multi-center prospective designs with continuous temperature monitoring to define precise optimal ranges and further investigate causality and underlying mechanisms.

## Electronic supplementary material

Below is the link to the electronic supplementary material.

Supplementary Material 1

## Data Availability

The datasets used and/or analyzed during the current study are available from the corresponding authors on reasonable request.
