# Supplementary material for: The impact of season, temperature, and direct normal irradiance on IVF pregnancy outcomes: a retrospective cohort study
Source: Int J Biometeorol. 2025 Jun 18;69(8):2053–65. doi: 10.1007/s00484-025-02951-2 (PMC12287234; doi:10.1007/s00484-025-02951-2)
Supplement: Supplementary file 1 — Supplementary Material 1 [file 484_2025_2951_MOESM1_ESM.docx]

**
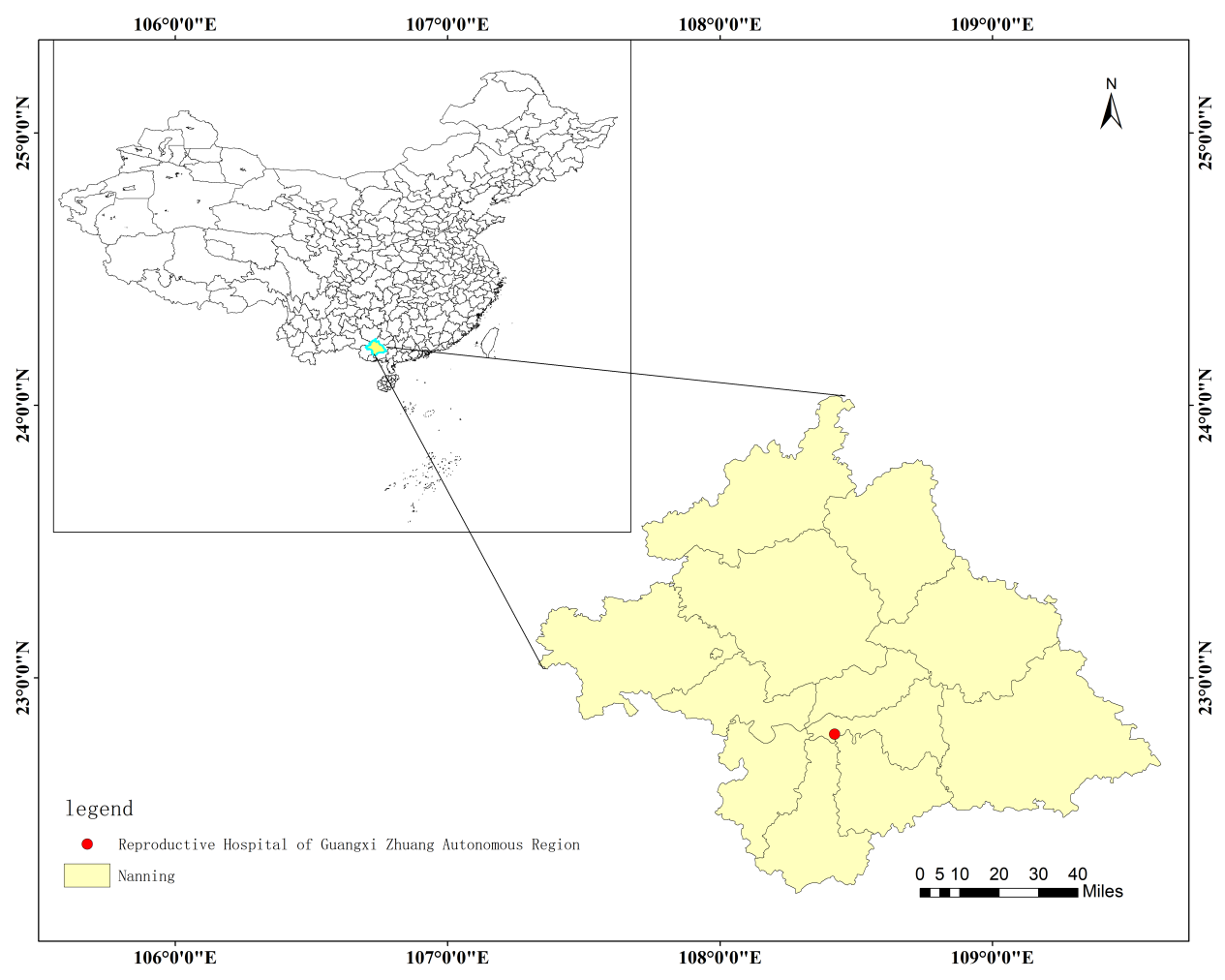
**

**Figure S1** Nanning meteorological data source areas and participant recruitment sites

**Table S1**. Descriptive statistics for demographics in the whole sample

| **Characteristics** | **Clinical pregnancy (n=562)** | **No clinical pregnancy(n=617)** | **Z/χ2** | ***P*** |
| --- | --- | --- | --- | --- |
| **Age** **(years)** | 34（31-37） | 36（33-40） | -7.907 | ＜0.001 |
| **Race** |  |  | 6.766 | 0.034 |
| Han | 281（50.00） | 281（45.54） |  |  |
| Zhuang | 248（44.13） | 313（50.73） |  |  |
| Others | 33（5.87） | 23（3.73） |  |  |
| **Occupation** |  |  | 2.428 | 0.297 |
| Employed | 255（45.37） | 271（43.92） |  |  |
| Agriculturial | 31（5.52） | 48（7.78） |  |  |
| Others | 276（49.11） | 298（48.30） |  |  |
| **Duration of infertility (years)** | 3.00（2.00-6.00） | 3.00（2.00-6.00） | -0.974 | 0.330 |
| **Type of infertility** |  |  | 0.773 | 0.379 |
| Primary | 241（42.88） | 249（40.36） |  |  |
| Secondary | 321（57.12） | 368（59.64） |  |  |
| **Infertility factor** |  |  | 18.830 | 0.003 |
| Tubal factor | 126（22.42） | 91（14.75） |  |  |
| Uterine factor | 3（0.53） | 4（0.65） |  |  |
| Female mixed factor | 170（30.25） | 179（29.01） |  |  |
| Male mixed factor | 23（4.09） | 38（6.16） |  |  |
| Both female and male factor | 236（41.99） | 294（47.65） |  |  |
| Diminished ovarian reserve | 4（0.71） | 6（0.97） |  |  |
| Unexplained | 0（0.00） | 5（0.81） |  |  |
| **FSH baseline (IU/L)** | 7.17（6.18-8.54） | 7.53（6.24-9.05） | -2.550 | 0.011 |
| **Protocol** |  |  | 6.510 | 0.011 |
| Long | 284（50.53） | 266（43.11） |  |  |
| Antagonist | 278（49.47） | 351（56.89） |  |  |
| **Gn dosage (IU)** | 2400（1800-3000） | 2625（2025-3375） | -3.687 | ＜0.001 |
| **Intimal thickness at trigger (mm)** | 10.30（9.00-11.60） | 9.90（8.60-11.30） | -2.943 | 0.003 |
| **F****ertilization method** |  |  | 2.293 | 0.130 |
| IVF | 470（83.63） | 495（80.23） |  |  |
| ICSI | 92（16.37） | 122（19.77） |  |  |
| **Day of transfer** |  |  | 21.713 | ＜0.001 |
| D3 | 330（58.72） | 442（71.64） |  |  |
| D5 | 232（42.28） | 175（28.36） |  |  |

Data are presented as median (interquartile range) for quantitative variables and n (%) for categorial variables.

FSH: follicle-stimulating hormone; Gn: gonadotropin; IVF: in vitro fertilization; ICSI: Intracytoplasmic Sperm Injection
